# Dominance and fitness costs of insect resistance to genetically modified *Bacillus thuringiensis* crops

**DOI:** 10.1080/21645698.2020.1852065

**Published:** 2020-12-31

**Authors:** Fangneng Huang

**Affiliations:** Department of Entomology, Louisiana State University Agricultural Center, Baton Rouge, LA, USA

**Keywords:** GM Bt crops, insect resistance management, dominance level, fitness costs of resistance

## Abstract

Evolution of resistance to genetically modified *Bacillus thuringiensis* (Bt) crops in pest populations is a major threat to the sustainability of the technology. Incidents of field resistance that have led to control problems of Bt crops or significantly reduced susceptibility of individual Bt proteins in pyramided plants have increased dramatically across the world, especially in recent years. Analysis of globally published data showed that 61.5% and 60.0% of the cases of resistance with major alleles that allowed homozygous resistant genotypes to survival on Bt crops were functionally non-recessive and did not involve fitness costs, respectively. Dominance levels (D_FL_s) measured on Bt plants ranged from −0.02 to 1.56 with a mean (± sem) of 0.35 ± 0.13 for the 13 cases of single-gene resistance to Bt plants that have been evaluated. Among these, all six cases with field control problems were functionally non-recessive with a mean D_FL_ of 0.63 ± 0.24, which was significantly greater than the D_FL_ (0.11 ± 0.07) of the seven cases without field resistance. In addition, index of fitness costs (IFC) of major resistance was calculated for each case based on the fitness of resistant (R’R’) and heterozygous (R’S’) genotypes on non-Bt plants divided by the fitness of their susceptible (S’S’) counterparts. The estimated IFCs for 15 cases of single-gene resistance were similar for R’R’ and R’S’, and for the cases with and without field resistance; and the values averaged 1.10 ± 0.12 for R’R’ and 1.20 ± 0.18 for R’S’. Limited published data suggest that resistance of insects to dual/multiple-gene Bt crops is likely to be more recessive than the related single-gene resistance, but their IFCs are similar. The quantitative analysis of the global data documents that the prevalence of non-recessive resistance has played an essential role in the widespread evolution of resistance to Bt crops, while the lack of fitness costs is apparently not as critical as the non-recessive resistance. The results suggest that planting of ‘high dose’ traits is an effective method for Bt crop IRM and more comprehensive management strategies that are also effective for functionally non-recessive resistance should be deployed.

## Introduction

The year of 1996 marked the first year that genetically modified (GM) crops expressing *Bacillus thuringiensis* (Bt) genes were officially commercialized in the US and several other countries.^1^ Since then, Bt crops have gained widespread acceptance throughout the world. By 2018, a total of more than one billion hectares of Bt crops had been planted in more than 20 countries.^1^ The predominant Bt crops planted are maize, cotton, and soybean. Bt crop traits are usually highly effective in controlling some major insect pests; thus, considerable economic, environmental, and social benefits have been gained from planting Bt crops in both industrial and developing countries.^1–5^

However, evolution of resistance in target insect populations is a great threat to the sustainability of the Bt crop technology.^6–8^ To delay resistance evolution, a ‘high dose/refuge’ (HDR) insect resistance management (IRM) strategy has been recommended in the U.S. and several other countries. ^9,10^ This strategy requires crop growers to plant a portion of the crop with ‘high dose’ Bt plants that can kill almost all homozygous-susceptible (SS) individuals and heterozygous-resistant (RS) individuals of the target pest species, while the remaining portion of the crop is planted with non-Bt plants to serve as a ‘refuge’ for SS populations. In this scenario, abundant SS individuals from the refuge plants will be available to mate with the rare homozygous-resistant (RR) survivors from the Bt plants. In this way, the majority of the offspring, if they possess resistance alleles, should be heterozygous, and these RS individuals will be killed by the ‘high dose’ Bt plants. As a result, resistance evolution should be greatly delayed.^9,11^ The current HDR for Bt crop IRM was largely based on knowledge generated from earlier laboratory studies of resistance to purified Bt proteins and Bt insecticides. Several earlier studies showed that high-level resistance to purified Bt proteins or Bt insecticides was typically recessive.^11–14^ In addition, it was also thought that Bt plants might be able to make ‘genetically’ non-recessive resistance functionally recessive because GM plants could be engineered to consistently express high levels of Bt proteins that would be capable of killing a large portion of ‘genetically’ non-recessive RS individuals. ^11,15^ Thus, functionally recessive resistance is a key foundation for the success of HDR strategy.^14,16,17^ Another IRM strategy for Bt crops is ‘gene pyramiding’ of two or more Bt genes with dissimilar modes of action.^18–20^ In the gene pyramiding, if individuals in insect populations carrying resistance alleles to one Bt protein are rare, the individuals simultaneously possess resistance alleles to two or more Bt proteins must be very rare. No significant cross-resistance that allows survival of resistant insects to one Bt protein on pyramided plants is a key assumption for the success of this strategy.

Fitness costs of resistance refers to a reduced fitness (e.g. delayed development, higher mortality, lower reproduction) of RR or RS individuals relative to SS individuals in the absence of selection. If both RR and RS individuals show fitness costs, the phenomenon is called non-recessive fitness costs. Otherwise, if only RR individuals show fitness costs, but RS performs similarly to SS, the fitness cost is considered recessive.^21–23^ Both the dominance level and fitness costs of resistance are important factors in resistance evolution. Fitness costs of resistance could result in declines in resistance and even reversion to susceptibility after selection pressure is removed.^21–23^ Earlier studies with purified Bt proteins or Bt insecticides showed that Bt resistance, especially high-level resistance, was often associated with fitness costs. ^12,23,24^ Thus, fitness costs are also considered a positive factor that may elevate the effectiveness of refuge planting for Bt crop IRM.^23^

After 20+ years of global Bt crop use, field resistance that has resulted in reduced efficacy of Bt crops or significantly reduced susceptibility of individual Bt proteins in pyramided Bt plants has been documented in at least 20 cases involving seven major pest species of maize and cotton in six countries across four continents.^6–8^ In addition, major resistance alleles that allow RR individuals to survive and complete their life cycle on Bt plants,^25^ while susceptible individuals of the species are controlled by the Bt plants, have also been isolated in several cases.^6,7^ To avoid any confusions with the survival due to natural tolerance, in this review, the term ‘major resistance allele’ for Bt plants is defined as RR individuals (populations, colonies, strains) possessing homozygous resistance alleles should exhibit a significantly greater rate to survive and complete their life cycles on the Bt plants, relative to their susceptible counterparts. It should be pointed out that there is variability in Bt susceptibility within populations of a same pest species even before Bt crops are commercialized. Information that was used to judge if a case of resistance qualified as ‘a major resistance allele’ in this review was based on only the resistant and susceptible insect populations used in the peer-reviewed studies. Based on this definition, ‘field resistance’ certainly qualifies as ‘major resistance’. However, finding major resistance alleles in a pest population does not necessarily indicate an immediate threat of field resistance to the Bt plants.^26^ Field resistance can occur when the frequency of major resistance alleles becomes sufficiently common to reduce efficacy of the Bt crop in field.^6,17^ During the last two decades, many studies have been conducted in the world to characterize the resistance (e.g. dominance, fitness costs, etc.) for some of these cases involving major resistance alleles including field resistance. Several previous reviews have analyzed the general conditions that are associated with the evolution of resistance to Bt proteins and/or Bt plants.^6,7,11,12,14,17,23,27–30^ In this mini review, I focus on only two important aspects that are closely related to resistance management: dominance level and fitness costs of resistance to Bt plants. More specifically, in the current review I will first extend the methods that are used to measure dominance levels/fitness costs of resistance in insect on single-gene Bt plants to pyramided Bt plants and then use the related globally published data to quantitatively analyze the relationship between dominance levels/fitness costs and the evolution of resistance to Bt crops. In addition, variations in the dominance levels/fitness costs among pest populations, Bt proteins, test methods, and crop-pest systems are also discussed. Knowledge generated from this analysis should be useful in understanding the reasons that have led to the recent wide occurrence of field resistance to Bt crops in the world and in refining current IRM strategies for the sustainable use of Bt crop technology.

## Criteria for literature selection and cases of resistance to Bt crops

Google Scholar (https://scholar.google.com/) was used to search the related literature published before January 31, 2020. Because Bt proteins expressed in GM plants can be different from the proteins produced by *B. thuringiensis* bacteria, information generated from the studies that used Bt protoxin, activated proteins, or microbial insecticides may not directly represent the status of insect resistance to Bt crops.^31^ To ensure data used in this review more accurately reflect the real situation of resistance to Bt crops, the following three criteria were used in selection of literature. First, only articles published in peer-reviewed journals were used. Second, resistant insects used in studies must have possessed major resistant genes to the Bt plants as defined above.^17,25^ Third, biological parameters measured in the studies must have been obtained from assays using whole plants/plant tissues of maize or cotton, because, by far, field resistance to Bt crops has been found only in Bt maize or Bt cotton.^7^ In other words, those studies using the ‘resistant’ populations that had not been documented to carry major resistance alleles to Bt plants, as well as data generated from assays on meridic diet or Bt protein-treated diet, or plants other than maize or cotton, were not included in this review. Use of published data was approved by the senior or corresponding author of each selected publication. To facilitate the analysis, the definition of a ‘case’ of resistance described in reference^7^ was adopted, which means that each case of single-gene resistance represents a resistance of one pest species in one country to one Bt protein in the crop plant. In addition, in this review, the definition of ‘case’ of single-gene resistance was also extended to include dual-/multiple-gene resistance. A case of resistance to dual/multiple-gene Bt gene plants means a resistance of one pest species in one country to the dual-/multiple-Bt proteins expressed in a pyramided crop trait. A pyramided crop trait is a GM plant product that contains two or more Bt genes with dissimilar modes of action for a target pest species.^32^

Based on the literature selection criteria described above, dominance level of resistance to Bt plants was evaluated in a total of 26 studies, which involved 17 cases of major resistance in seven target insect species to eight Bt proteins in maize or cotton (Table 1 and Supporting information: Table A1). The seven insect species comprised almost all global major target pests of Bt maize and Bt cotton, and the eight Bt proteins included almost all the Bt proteins expressed in Bt crops currently available in the global market. Among the 17 cases, 13 cases were resistant to single-gene Bt crops and the rest four were associated with resistance to dual/multiple-gene Bt crops. Resistance accompanied by field control problems, defined as field resistance mentioned above, has been reported in six of the 13 cases of single-gene resistance. The term ‘field resistance’ in this review means that the resistance has resulted in field control problems of a Bt crop, or significantly reduced susceptibility of individual Bt proteins in pyramided Bt plants, which is similar to the ‘practical resistance’ defined in the reference.^6^ Besides the condition of field control problem, the criteria for ‘practical resistance’ also include that >50% of individuals in a population are resistant.^6,60^ Because resistance allele frequencies for some cases that qualify as ‘field resistance’ as described above have not been reported, or the rate of the resistant individuals for some cases was <50%, the term ‘field resistance’ is used in this review. The six cases with field resistance are the resistance of *Busseola fusca* to Cry1Ab maize in South Africa^61^; *Spodoptera frugiperda* to Cry1F maize in Brazil^59^ and in the U.S.^62,63^; *Diabrotica virgifera virgifera* to Cry3Bb1 maize in the U.S.^64^; *D. virgifera virgifera* to eCry3.1Ab maize in the U.S.^65^; and *S. frugiperda* to Cry1A.105 maize in the U.S.^63,66^ The documented high resistance allele frequency in *S. frugiperda* to Cry1A.105 maize and the observed high cross-resistance of the insect between Cry1F and Cry1A.105 maize^63,66^ were similar to the results reported in the resistance of *Diatraea saccharalis* to Cry1A.105 maize in Argentina^67^ which was listed as a case of ‘practical resistance’ in the reference.^7^ Thus, the resistance of *S. frugiperda* to Cry1A.105 maize in the U.S was also considered a case of field resistance in the current review. Major resistance of the seven cases without field control problems was usually isolated through laboratory selections. Three of the seven laboratory cases were established using massive-selections, while the rest four were isolated with F_2_ screen. As mentioned above, these laboratory selections may not exactly reflect the real situation of the field selections, especially for those cases established from long-term and massive-selections on Bt protein-treated diet.^31^ Thus, it is possible that the selection methods might confound the analysis in this review.

**Table 1.** Dominance levels (D_FL_s) of 17 cases of major resistance to Bt crops in seven target pest species

Case of resistance	Field resistance	No. populations	D_FL_	Reference
**Resistance to single-gene Bt crops**				
*B. fusca* to Cry1Ab maize in S. Africa	Yes	1	1.56	^33^
*S. frugiperda* to Cry1F maize in Brazil	Yes	5	0.23	^34–36^
*S. frugiperda* to Cry1F maize in U.S.	Yes	2	0.10	^37,38^
*D. virgifera virgifera* to Cry3Bb1 maize in U.S.	Yes	5	0.41	^39–41^
*D. virgifera virgifera* to eCry3.1Ab maize in U.S.	Yes	1	1.16	^42^
*S. frugiperda* to Cry1A.105 maize in U.S.	Yes	2	0.34	^43^
*O. nubilalis* to Cry1F maize in U.S.	No	1	0.04	^44^
*S. frugiperda* to Cry2Ab2 maize in U.S.	No	1	−0.02	^45^
*S. frugiperda* to Vip3A maize in Brazil	No	1	0.00	^46,47^
*S. frugiperda* to Vip3A maize in U.S.	No	1	0.00	^48^
*H. armigera* to Cry1Ac cotton in Australia	No	2	0.33	^49,50^
*P. gossypiella* to Cry1Ac cotton in U.S.	No	1	0.00	^51^
*D. saccharalis* to Cry1Ab maize in U.S.	No	1	0.41	^32,52,53^
**Resistance to dual/multiple-gene Bt crops**				
*S. frugiperda* to Cry1A.105/Cry2Ab maize in Brazil	No	2	0.00	^54,55^
*S. frugiperda* to Cry1A.105/Cry2Ab maize in U.S	No	1	0.20	^56,57^
*S. frugiperda* to Cry1Ab/Vip3A maize in Brazil	No	1	0.00	^54^
*S. frugiperda* to Cry1A.105/Cry2Ab2/Cry1F in Brazil	No	1	0.00	^54,58^

On the other hand, fitness costs of resistance to Bt plants have been investigated in a total of 28 studies in the world, which involved 20 cases of major resistance in eight insect species to eight Bt proteins in maize or cotton (Table 2 and Supporting information: Table A2). The eight insect species included all the seven species described above in which the dominance level of resistance has been investigated, plus *Trichoplusia ni*, a secondary target species of Bt cotton in the U.S. In addition, the eight Bt proteins are the same as those evaluated in the studies of dominance levels. Among the 20 cases, 15 cases involved single-gene resistance and five cases were associated with resistance to dual/multiple-gene Bt plants. The 15 cases of single-gene resistance also included all the six cases with field resistance mentioned above, while field resistance has not been documented for all other cases.

**Table 2.** Index of fitness costs (IFCs) of 20 cases of major resistance to Bt crops in eight target pest species

Case of resistance	Field resistance	No. populations investigated	IFC_R’R’_ ^a^	IFC_R’S’_	Reference
**Resistance to single-gene Bt crops**					
*B. fusca* to Cry1Ab maize in S. Africa	Yes	1	1.91	n/a	^68^
*S. frugiperda* to Cry1F maize in Brazil	Yes	4	0.94	0.98	^35,36,54^
*S. frugiperda* to Cry1F maize in U.S.	Yes	3	0.68	0.94	^69,70^
*D. virgifera virgifera* to Cry3Bb1 maize in U.S.	Yes	8	1.08	n/a	^40,41,71–73^
*D. virgifera virgifera* to eCry3.1Ab maize in U.S.	Yes	1	1.64	n/a	^74^
*S. frugiperda* to Cry1A.105 maize in U.S.	Yes	2	1.65	1.99	^43^
*O. nubilalis* to Cry1F maize in U.S.	No	1	0.77	0.96	^75^
*S. frugiperda* to Cry2Ab2 maize in U.S.	No	1	1.87	2.39	^45^
*S. frugiperda to Vip3A maize in Brazil*	No	1	0.80	0.95	^46^
*S. frugiperda* to Vip3A maize in U.S.	No	1	1.03	1.06	^76^
*H. armigera* to Cry1Ac cotton in Australia	No	2	0.71	0.95	^49,77^
*H. armigera* to Cry1Ac cotton in China	No	1	0.77	n/a	^78^
*P. gossypiella* to Cry1Ac cotton in U.S.	No	1	0.48	0.52	^51^
*D. saccharalis* to Cry1Ab maize in U.S.	No	2	1.28	1.23	^32,52,53^
*T. ni* to Cry1Ac cotton in U.S.	No	1	0.94	n/a	^79^
**Resistance to dual/multiple-gene Bt crops**					
*S. frugiperda* to Cry1A.105/Cry2Ab maize in Brazil	No	2	1.00	1.05	^54,55^
*S. frugiperda* to Cry1A.105/Cry2Ab maize in U.S	No	1	0.73	1.15	^56,57^
*T. ni* to Cry1Ac/Cry2A cotton in U.S.	No	1	0.81	n/a	^79^
*S. frugiperda* to Cry1Ab/Vip3A maize in Brazil	No	1	0.86	1.08	^54^
*S. frugiperda* to Cry1A.105/Cry2Ab2/Cry1F in Brazil	No	2	1.00	0.98	^54,58^

^a^Developmental delay could be a more relevant fitness factor for pests that have multiple generations per cropping cycle than univoltine insects. Six of the 20 cases listed in the table involved the use of the parameter ‘insect developmental time’ in estimation of fitness costs. Except for the case related to the resistance of *D. virgifera virgifera* to Cry3Bb1 maize, all other five cases were associated with insects having multiple generations per year. For the resistance of the univoltine *D. virgifera virgifera* to Cry3Bb1 maize, two of the five related studies considered ‘days of development to adults’ in the fitness calculations. Because the developmental time on non-Bt plants reported in the references^40,41^ was almost same between RR and SS, IFC_R’R’_ estimated with/without considering the parameter ‘developmental time’ was virtually identical (data not shown). To be consistent, the method described in the text was used to calculate IFCs for all the cases regardless of the number of generations per cropping season for the insect evaluated.

## Measurement and calculation of dominance levels of resistance to Bt plants

As described in the reference^14^, dominance of a single gene resistance can be measured in three ways: dominance of insecticide resistance (e.g. D_LC_), which is based on the dose-mortality response curves of RR, RS and SS genotypes; effective dominance (D_ML_), which is based on the mortality levels of the three genotypes at a given toxin concentration; and dominance of relative fitness in the treated area (D_WT_), which is based on the fitness of the three genotypes at a given toxin concentration. These three measurements are related, but they are not the same. Among the three, D_WT_ provides the most useful information for resistance management.^14^ However, measurement of D_WT_ is usually more difficult than measurements of D_LC_ and D_ML_. For this reason, most of the early studies of Bt resistance measured only D_LC_ or D_ML_. In this study, I extend the methods for calculating the dominance levels of single-gene resistance described in the reference^14^ to also include the cases of resistance to dual/multiple-gene Bt plants. More specifically, the dominance for single- or dual/multiple-gene resistance to Bt plants can be calculated as:

$$D{\;}_{ML}^{\prime }=\left(M_{R^{\prime }S^{\prime }}-M_{S^{\prime }S^{\prime }}\right)/\left(M_{R^{\prime }R^{\prime }}-M_{S^{\prime }S^{\prime }}\right)\;\;or\;D{\;}_{WT}^{\prime }=\;\left(W_{TR^{\prime }S^{\prime }}-W_{TS^{\prime }S^{\prime }}\right)/\left(W_{TR^{\prime }R^{\prime }}-W_{TS^{\prime }S^{\prime }}\right)$$

Here, D’_ML_ is the effective dominance of single- or dual/multiple-gene resistance to Bt plants based on the mortality levels of the three genotypes (R’R’, R’S’, and S’S’) on Bt plants; and D’_WT_ is the dominance of relative fitness in the treated area based on the fitness of the three genotypes on Bt plants. M_R’R’_, M_R’S’_, and M_S’S’_ are the mortality levels of the single- or dual/multiple-gene homozygous-resistant (R’R’), heterozygous (R’S’), and homozygous-susceptible (S’S’) genotypes on the corresponding single- or dual/multiple-gene Bt plants, respectively. For examples, If A, B, and C represent three different resistant alleles and a, b, and c refers to the three corresponding susceptible alleles of the three genes, R’R’, R’S’, and S’S’ represent AA, Aa, and aa for a single-gene resistance; AABB, AaBb, and aabb for a dual-gene resistance; or AABBCC, AaBbCc, and aabbcc for a triple-gene resistance. The measurement of D’_ML_ or D’_WT_ described here can also be used to calculate the dominance levels for other genotypes in dual/multiple-gene resistance (e.g. AABb, AaBB, AABBCc, etc.) as described in reference.^56^ Among the 22 studies that evaluated the dominance level of resistance to single-gene Bt crops, five studies measured D’_WT_, while the other 17 measured D’_ML_s that were based on survivorship of S’S’, R’S’, and R’R’ individuals on whole Bt plants or plant tissues using exposure times from 7 d to a period encompassing neonate-to adult development (Table A1). Among the five studies that evaluated dominance level of dual/multiple-gene resistance to Bt plants, one study evaluated D’_WT_ and the other four measured D’_ML_ (Table A1).

In this review, dominance level (functionally) (hereafter referred to as D_FL_) was calculated for each case of single- or dual/multiple-gene resistance to Bt plants based on the values of D’_ML_ or D’_WT_ reported in each study. Similarly, as described in the reference^14^, D_FL_ values normally vary from 0 to 1 (D_FL_ = 0, functionally completely recessive; D_FL_ = 1, functionally completely dominant). In the situations in which >1 study was conducted, >1 insect population was evaluated, or >1 trial was performed for a case, the D_FL_ for the case was calculated as the average of D’_ML_s or D’_WT_s, or the mixed D’_ML_s and D’_WT_s across studies, populations, or trials. Data sources and calculations of D_FL_s of the 17 cases are listed in the Supporting Information (Appendix Table A1) linked to this publication.

## Measurement and calculation of index of fitness costs of resistance to Bt plants

To facilitate quantitative analysis of the fitness costs of resistance to Bt plants, a term, index of fitness cost (IFC), is used in this review. IFC for both single- and dual/multiple-gene resistance is calculated using the formula^81^:

$$IFC_{R^{\prime }R^{\prime }}=F_{R^{\prime }R^{\prime }}/F_{S^{\prime }S^{\prime }}\;and\;IFC_{R^{\prime }S^{\prime }}=F_{R^{\prime }S^{\prime }}/F_{S^{\prime }S^{\prime }}$$

Here IFC_R’R’_ and IFC_R’S’_ refer to the index of fitness costs of resistant-homozygous (R’R’) and – heterozygous (R’S’) genotypes, respectively. R’R’, R’S’, and S’S’ represent the three genotypes as described in the measurement of D’_ML_ or D’_WT_. F_S’S’_, F_R’S’_, and F_R’R’_ refer to the fitness of S’S’, R’S’, and R’R’ genotypes on non-Bt plants or non-Bt plant tissues, respectively. IFC < 1 means that fitness costs are associated with the resistance; IFC = 1 suggests lack of fitness costs; and IFC > 1 indicates that there are fitness advantages. If IFC_R’R’_ < 1 but IFC_R’S’_ = 1 for a resistance, fitness costs are recessive, while if both IFC_R’R’_ and IFC_R’S’_ are < 1, fitness costs are non-recessive. Non-recessive fitness costs are considered more important in resistance management than recessive fitness costs, because R’S’ individuals are usually much more abundant than R’R’ individuals in the absence of Bt selection.^23^ Similarly as mentioned for D’_ML_ or D’_WT_, the measurement of IFC described here could also be used to calculate the fitness costs of other genotypes in dual/multiple-gene resistance to Bt crops (e.g. AaBB, AABbCc, etc.).

In the review of the 28 studies, only one^71^ reported the IFC value directly, while all others showed various fitness parameters. These biological parameters included insect survivorship with a wide range of exposure period, insect development, growth (e.g. larval and/or pupal body mass), sex ratio, egg production, and egg hatching rate (Supporting Information Appendix Table A2). One study evaluated fitness for both field-collected parental (F0) and F1 generations.^68^ In this review, a ‘combined fitness index’ was used to measure the fitness (F_S’S’_, F_R’S’_, or F_R’R’_) of each insect genotype on plants or plant tissue. Combined F_S’S’_, F_R’S’_, or F_R’R’_ values were calculated based on the most comprehensive measurements reported in each study with the methods described below:

- If the intrinsic rate of population increase, r_m_, was available in a study, r_m_ was used as the combined fitness index and no other parameters were considered in IFC calculation for the study.
- If r_m_ was not available, but insect survivorship, developmental time, egg production, and egg hatching rate were reported, the combined fitness index was calculated as: (insect survivorship x egg production x egg hatching rate)/insect developmental time. No other parameters were considered in IFC calculation for the study.
- If any of the parameters in the formula described in b was not available, the item for that parameter was excluded in calculation of the combined fitness index.

IFC was calculated for both R’R’ and R’S’ (if data available) for each population in each study. Similarly, as described for the D_FL_ calculation, in situations in which >1 study was conducted, >1 insect population was evaluated, or >1 trial was performed for a case, the IFC of the case was calculated as the average of IFCs across studies, populations, or trials. Data sources and detailed IFC calculations for the 20 cases are listed in the Supporting Information (Appendix Table A2).

## Dominance, D_FL_, of Resistance to Bt crops

Analysis of global studies showed that D_FL_s of the 13 cases of major resistance to single-gene Bt crops ranged from −0.02 to 1.56 with a mean of 0.35 ± 0.13 (Table 1). Among the 13 cases, functionally recessive resistance was reported in only five cases (or 38.5% of the total) with a D_FL_ of zero or close to zero. These five cases were the resistance of *Ostrinia nubilalis* to Cry1F maize in the U.S. (D_FL_ = 0.04), *S. frugiperda* to Cry2Ab2 maize in the U.S. (D_FL_ = −0.02), *S. frugiperda* to Vip3A maize in Brazil and the U.S. (D_FL_ = 0 for both cases), and *Pectinophora gossypiella* to Cry1Ac cotton in the U.S. (D_FL_ = 0). To date, field resistance has not been reported for any of these five cases. Resistance in the other eight cases (61.5%) was functionally non-recessive with a D_FL_ of 0.10 or greater (Table 1).

All of the six cases with field resistance were functionally non-recessive with a D_FL_ ranging from 0.10 to 1.56 (Table 1). The resistance in two of the six cases was completely or even over-completely dominant: resistance in *B. fusca* to Cry1Ab maize in South Africa, with a D_FL_ of 1.56, and *D. virgifera virgifera* to eCry3.1Ab maize in the U.S. with a D_FL_ of 1.16. Resistance in the other four cases of field resistance ranged from incompletely recessive to co-dominant. The mean D_FL_ for the six cases with field resistance was 0.63 ± 0.24 (mean ± sem), while it was 0.11 ± 0.07 for the seven cases without practical field resistance (Table 3). The difference in D_FL_s between the cases with and without field resistance was significant (SAS PROC NPAR1WAY Wilcoxon, *P* = .0309) (Table 3). The four cases of dual/multiple-gene resistance in which D_FL_ has been evaluated involved only the resistance of *S. frugiperda* to Bt maize in Brazil and the U.S. These limited data suggest that dual/multiple-gene resistance is more likely to be recessive than the related single-gene resistance. Three of the four dual/multiple-gene resistance cases were functionally recessive with a D_FL_ of zero and the remainder was incompletely recessive with a D_FL_ of 0.20 (Table 1).

**Table 3.** Comparison of dominance levels (D_FL_s) and index of fitness costs (IFC) of single-gene resistance between cases with and without field resistance to Bt crops

			Index of fitness costs			
	Dominance level		R’R’		R’S’	
Resistance status	No. case	D_FL_	No. case	IFC_R’R’_	No case	IFC_R’S’_
Cases with field resistance occurred	6	0.63 ± 0.24	6	1.32 ± 0.20	3	1.30 ± 0.34
Cases with field resistance not occurred yet	7	0.11 ± 0.07	9	0.96 ± 0.14	7	1.15 ± 0.22
Wilcoxon non-parametric test	*P* = .0309		*P* = .1941		*P* = 1.000	

As mentioned above, because it is usually difficult to measure D’_WT_, 10 of the 13 single-gene resistance cases actually measured only D’_ML_s, which is calculated based on mortality only. It is believed that the dominance level can be over-estimated using only D’_ML_, because R’S’ survivors can be less fit than R’R’ survivors.^14^ GM Bt plants are usually very effective against S’S’ and thus S’S’ individuals rarely survive on Bt crops. In this situation, if R’S’ survivors are less fit than R’R’ survivors, the actual D’_WT_ will be lower than D’_ML_. However, data from the 13 cases of single-gene resistance analyzed in this review do not provide any evidence to indicate that D’_ML_ is greater than D’_WT_. For example, both D’_ML_ and D’_WT_ were estimated for the Brazilian case of Cry1F resistance in *S. frugiperda*. Based on neonate-to-adult survivorships on Cry1F leaf tissue, Farias *et al*.^34^ reported a D’_ML_ of 0.15 for the population BR25R. In another study, Leite *et al*.^35^ assessed the dominance levels of two populations (IrmaF and IrmaD) based on a ‘fitness index’ on Cry1F maize leaf tissue. The ‘fitness index’ was calculated using the formula, fitness index = (neonate-to-pupal survival x pupal weight)/neonate-to-pupal development time. Using this method, the dominance levels for IrmaF and IrmaD were estimated to be 0.36. In addition, Santos-Amaya *et al*.^36^ also examined the dominance levels of two other populations (MTH and MRH) on Cry1F plants using the same ‘fitness index’ as described in reference.^35^ The estimated dominance levels for MTH and MRH were 0.12 and 0.17, respectively. Studies have shown that pupal body weight is usually highly correlated to reproduction in many lepidopteran species.^82^ Thus, the estimated dominance levels in the four populations evaluated in references^35,36^ could be considered a close estimate to the true D_WT._ The average dominance level (or D’_WT_) of the four populations was 0.25, which was somewhat greater than the D’_ML_ (0.15) estimated in reference.^34^ In addition, three of the five cases of single-gene resistance that were identified to be completely recessive or nearly completely recessive were based on the measurement of D’_ML_s. More importantly, the significantly greater overall D_FL_ values for the six cases with field resistance, relative to the seven cases without field resistance, are particularly telling and document that D_FL_s estimated in these studies were closely correlated to the resistance evolution in the field.

There were a few cases in which D_FL_ of a case have been evaluated for multiple populations. In some cases, D_FL_ values among populations within a case were consistent. For example, the resistance of *S. frugiperda* to Cry1F maize was incompletely recessive in all five Brazilian populations examined (Table A1). Similarly, the resistance of *D. virgifera virgifera* to Cry3Bb1 maize in five U.S. populations was all incompletely recessive or codominant with D_FL_ values ranging from 0.27 to 0.59 (Table A1). However, in some cases, notable variations were observed. For example, the resistance of *Helicoverpa armigera* to Cry1Ac cotton in Australia was completely recessive on 4-week old cotton,^49^ while it was incompletely dominant on 14-week cotton.^50^ Variations in D_FL_s among populations in a case were also observed for the resistance of *S. frugiperda* to Cry1F maize and Cry1A.105 maize in the U.S. (Table A1).^37,38,43^ In addition, differences in D_FL_s were noted among cases of a same pest-Bt crop system, but the differences were relatively small. For example, both cases of *S. frugiperda* resistance to Cry1F maize in Brazil and the U.S. were incompletely recessive. Similarly, both cases of resistance to Vip3A maize were completely recessive in the two countries. In contrast, variation in D_FL_s for a target pest species appeared to be greater among different Bt protein-crop systems. For example, D_FL_s of *S. frugiperda* resistance varied from −0.02 on Cry2Ab2 maize to 0.34 on Cry1A.105 maize, and D_FL_s of *D. virgifera virgifera* resistance differed from 0.41 on Cry3Bb1 maize to 1.16 on eCry3.1Ab maize. Nevertheless, the published data showed that all three cases of resistance associated with maize plants expressing the Vip3A protein were completely recessive with a D_FL_ of zero which provides evidence that GM plants containing Vip3A gene most likely produce the necessary ‘high dose’ as required for the HDR strategy. The observed variation in D_FL_s among populations or among cases within the same species could be due to genetic differences in resistant genes and/or differences in test conditions, such as differences in plant growth stages or tissues used in bioassays. Thus, experiments testing with multiple insect populations under different environmental conditions are necessary in order to generate robust D_FL_s.

It should be pointed out that the estimated dominance of resistance for dual/multiple- gene resistance to Bt plants may not only reflect the inheritance of survival/fitness to each Bt protein in a pyramid, but also can be associated with the interactions of different Bt proteins in plants, such as effects of cross-resistance and the extent of redundant killing. Exploring such relations in detail is beyond the scope of this review. Nevertheless, information on the dominance of dual/multiple-gene resistance to Bt plants should also be useful in resistance management as for the single-gene resistance. Additional studies are necessary to analyze the dominance levels of dual-/multiple-gene resistance to Bt plants. However, the lower dominance levels observed from the limited cases of dual-/multiple-gene resistance relative to single-gene resistance are an encouraging sign for the use of pyramided Bt crop traits for IRM.^10^ The results suggest that pyramiding with dissimilar Bt proteins could make a non-recessive resistance to single-gene Bt plants functionally more recessive. In the U.S., single-gene Bt cotton has already been completely phased out of the market and replaced by pyramided varieties. Pyramided Bt maize was first commercialized in 2010 and since then pyramided Bt maize traits have been widely planted in the U.S. and several other countries. However, individual Bt proteins in all current pyramided crop traits have been used sequentially. In the sequential use of Bt proteins, there is possibility as only one active Bt gene being introduced in each ‘new pyramided trait’ if the target insects already become resistant to all other Bt proteins after being used for many years. In such cases, a new ‘pyramided trait’ essentially functionally just likes a single-gene trait, which would dramatically reduce the effectiveness of pyramiding for IRM.^6,81,83–86^ It is believed that sequential use of Cry1 than Cry1 + Cry2 proteins in Bt maize and cotton could be a key factor that has contributed to the recent widespread occurrence of the field resistance of *H. zea* to pyramided Cry1A/Cry2A maize and cotton in the U.S. and the field resistance of *P. gossypiella* to Cry1A/Cry2A cotton in India.^80^

## Fitness costs, IFCs, of resistance to Bt crops

Global data analysis of the 28 studies showed that only six (or 40.0% of the total) of the 15 cases of major resistance to single-gene Bt crops were likely associated with fitness costs (Table 2). These six cases were the resistance of *S. frugiperda* to Cry1F maize in the U.S. (IFC_R’R’_ = 0.68), *O. nubilalis* to Cry1F maize in U.S. (IFC_R’R’_ = 0.77), *S. frugiperda* to Vip3A maize in Brazil (IFC_R’R’_ = 0.80), *H. armigera* to Cry1Ac cotton in Australia (IFC_R’R’_ = 0.71) and China (IFC_R’R’_ = 0.77), and *P. gossypiella* to Cry1Ac cotton in U.S. (IFC_R’R’_ = 0.48) (Table 2). Five cases (33.3%) of single-gene resistance showed some level of fitness advantage; these were the resistance of *B. fusca* to Cry1Ab maize in South Africa (IFC_R’R’_ = 1.91), *D. virgifera virgifera* to eCry3.1Ab maize in the U.S. (IFC_R’R’_ = 1.64), *S. frugiperda* to Cry1A.105 (IFC_R’R’_ = 1.65) and Cry2Ab2 (IFC_R’R’_ = 1.87) maize in the U.S., and *D. saccharalis* to Cry1Ab maize in the U.S. (IFC_R’R’_ = 1.28). The rest of the four cases (33.3%) exhibited IFC_R’R’_ values from 0.94 to 1.08, indicating lack of fitness costs or advantages. The 15 cases of single-gene resistance had a mean IFC_R’R’_ of 1.10 ± 0.12 (mean ± sem) and the IFC_R’R’_ (1.32 ± 0.20) of the six cases with field resistance was statistically similar to that (0.96 ± 0.14) of the nine cases without field resistance (SAS PROC NPAR1WAY Wilcoxon, *P* = .1941) (Table 3).

**Figure 1.**
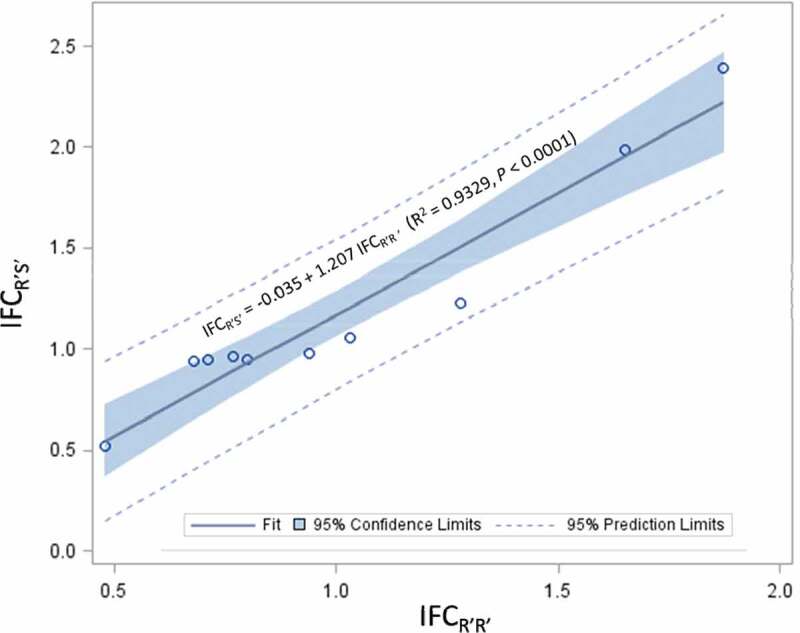
Correlation analysis on index of fitness costs (IFC) of ten single-gene major resistance cases to single-gene Bt plants between resistant-homozygous (R’R’) and -heterozygous (R’S’) genotypes. Analysis was performed by treating the index of fitness costs for R’R’ (IFC_R’R’_) of a case as the independent variable and the index of fitness costs for R’S’ (IFC_R’S’_) of the case as the dependent variable

In addition, fitness costs of R’S’ were also evaluated for 10 of the 15 single-gene resistance cases, which included three cases with field resistance and seven cases without field resistance (Table 2). Fitness costs of R’S’ were clearly observed in only one of the ten cases, which was the resistance of *P. gossypiella* to Cry1Ac cotton in the U.S. with an IFC_R’S’_ of 0.51. In contrast, R’S’ individuals in three cases had a greater fitness than SS individuals; these cases were the resistance of *S. frugiperda* to Cry1A.105 (IFC_R’S’_ = 1.99) and Cry2Ab2 (IFC_R’S’_ = 2.39) maize and *D. saccharalis* to Cry1Ab maize (IFC_R’S’_ = 1.23) in the U.S. IFC_R’S’_ values of the remaining six cases ranged from 0.94 to 1.06, suggesting lack of fitness costs for R’S’. The mean IFC_R’S’_ (1.30 ± 0.34) of the three cases with field resistance was not different compared to that (1.15 ± 0.22) of the seven cases without field resistance (SAS PROC NPAR1WAY Wilcoxon, *P* = 1.000) (Table 3). IFC_R’S’_ is not independent of IFC_R’R’_; analysis of the ten cases in which both R’R’ and R’S’ were available showed a strong linear relationship between the two indices (IFC_R’S’_ = −0.035 + 1.207 IFC_R’R’_; *R*^2^ = 0.9329, *P* < .0001) (Figure 1). In addition, a paired *t*-test with ‘case’ as the subject factor also showed that the mean IFC_R’S’_ (1.20 ± 0.18) was significantly greater than the IFC_R’R’_ (1.02 ± 0.14) for the ten cases (*t* = −3.23, df = 9, *P* = .0103).

Fitness costs of dual/multiple-gene resistance were evaluated for only five cases and involved only two pest species, *S. frugiperda* and *T. ni* (Table 2). Fitness costs were likely associated with R’R’ in three of the five cases, which were the resistance of *S. frugiperda* to Cry1A.105/Cry2Ab2 maize in the U.S. (IFC_R’R’_ = 0.73) and to Cry1Ab/Vip3A maize in Brazil (IFC_R’R’_ = 0.86), and *T. ni* to Cry1Ac/Cry2A cotton in the U.S. (IFC_R’R’_ = 0.81). Fitness costs were not associated with the remaining two cases (IFC_R’R’_ = 1.0 for both cases). The mean IFC_R’R’_ for the five cases of dual-/multiple-gene resistance was 0.88 ± 0.05. IFC_R’S’_ has been evaluated for four of the five cases of dial/multiple-gene resistance and none of the four cases showed any fitness costs with an IFC_R’S’_ ranging from 0.98 to 1.15 and an average of 1.07 ± 0.04 (Table 2).

Fitness costs of resistance can vary depending on Bt protein, crop, insect species and population, test conditions, etc.^23^ As observed for the D_FL_s, variations in IFCs were also reported among populations and cases within the same pest-crop system. For example, in the Brazilian Cry1F-resistant populations, fitness costs were likely associated with the MRH population on non-Bt maize in the study^36^ and a population on non-Bt cotton evaluated in the reference^54^, while lack of fitness costs was observed for other populations and test conditions (Table A1). Similarly, notable fitness costs were observed in two U.S. Cry3Bb1-resistant populations of *D. virgifera virgifera*, but lack of fitness costs was detected for other populations (Table A1). In addition, fitness costs were observed in the resistance of *s, frugiperda* to Vip3A maize for both the Brazilian and U.S. populations on non-Bt maize plants, but not for the U.S. population on non-Bt cotton.^46,76^ In contrast, in some cases, fitness costs were consistent among populations within a pest-crop system. For examples, all of the three U.S. Cry1F-resistant populations of *S. frugiperda* exhibited some level of fitness costs (Table A1). Considerable fitness costs were also observed in the two Australian Cry1Ac-resistant populations of *H. armigera*. In addition, both U.S. Cry1A.105-resistant populations of *S. frugiperda* studied in the reference^43^ performed similarly on non-Bt maize and both did not show any fitness costs.

The significant number (33.3%) of cases of single-gene resistance with fitness advantages observed in this review is a surprise. Fitness advantage of Bt resistance has been supposed to be very rare.^87^ However, it is also believed that selection for Bt resistance could be linked to some genes that are favorable for insect growth and development.^72,87^ In addition, some of the comparisons might involve the use of unrelated resistant and susceptible strains that may have differed for reasons unrelated to resistance. Unfortunately the available data listed in Tables 1 and Tables 2 could not clarify the situation. Further studies are warranted to understand the biological mechanisms or other factors behind the observed ‘fitness advantages’ of Bt resistance. The greater IFC for RS than RR in the ten cases of single-gene resistance is not surprised, because the fitness costs in four of five cases were recessive. In addition, in the two cases of *S. frugiperda* resistance to Cry1A.105 and Cry2Ab2 maize in which the resistance showed a fitness advantage (IFC_RR_ > 1), the IFC_RS_s were still somewhat greater than the corresponding IFC_RR_s (Table 2). A possible reason for the greater performance of the RS relative to both RR and SS may be hybrid vigor, which could occur when crossing two populations that were inbred and had different genetic backgrounds.^23^ Differences between resistant and susceptible strains of an insect species that are unrelated to the Bt resistance could be caused by many factors such as sources of insect strains, lab adaptation, diet adaptation, or isolation in resistance selections. To ensure a similar genetic background between SS and RR, the RR populations used in the two studies had been backcrossed with SS for at least two times and then reselected for resistance before they were used for the crosses to generate RS genotypes. If the better performance of the RS genotypes reported in the two cases was truly caused by hybrid vigor, it suggests that additional backcrossing would be necessary to ensure a more similar genetic background between RR and SS to avoid any possible confounding effect of ‘hybrid vigor’. The use of a susceptible comparator with different genetic bases could undermine the importance of fitness costs in the field.^23^ In addition, choice of susceptible insect strain could also affect estimation of dominance because hybrid vigor would artificially inflate the apparent fitness of heterozygotes on Bt plants, as well as on non-Bt plants, and therefore could affect the calculated functional dominance values. However, a linear regression analysis (SAS PRO REG) with the ten cases that both IF_R’S’_ (x) and D_FL_ (y) data are available failed to find any linkage between the fitness advantage of RS and the functional dominance levels of the resistance (y = 0.106 + 0.031 x, correlation coefficient *R* = 0.10 (*P* = .7762)). Nevertheless, as described above, it is critical in study of fitness costs and dominance levels of resistance to ensure a similar genetic basis among insect populations. A common method used to achieve similar genetic bases is to backcross the resistant populations to their susceptible comparator and reselect the resistance in the backcrossed populations. Theoretically, the similarity in genetic background among insect populations increases as the number of back-crosses increases.

## Conclusion

It should be noted that major resistance genes for some insect-Bt crop systems, such as resistance of *O. nubilalis* to Cry1Ab maize, have not been identified yet. However, the 13 (for D_FL_) and 15 (for IFC) cases of major resistance to single-gene Bt crops analyzed in this review have included almost all the global major target pest species and all Bt proteins expressed in the world market of GM Bt crops. The surprisingly high rates of functionally non-recessive resistance (61.5%) and lack of fitness costs (60.0%) of resistance reported in this review clearly documented that high levels of resistance to Bt crops are usually non-recessive with no fitness costs. Use of insect populations with similar genetic background is critical in study of fitness costs and dominance levels of resistance. Otherwise, use of a susceptible comparator with different genetic background could undermine the importance of fitness costs in the field. Limited available data suggest that dual/multiple-gene Bt resistance is likely to be more recessive than the related single-gene resistance, but their IFCs are similar. Many factors can influence the speed of resistance development, but the documentations that all six cases of field resistance are functionally non-recessive, as well as the significantly greater D_FL_s of the cases with field resistance than those without field resistance provide clear evidence that the prevalence of non-recessive resistance has certainly played an essential role in the widespread occurrence of field resistance to Bt crops. In addition, the documented high rate of non-recessive resistance also provides solid counterevidence against a general application of the assumption of functionally recessive resistance for the recommended HDR strategy, at least for single-gene Bt crops. The lack of fitness costs might be associated with the widespread of the field resistance. However, the similar IFCs observed between the cases with and without field resistance suggest that the role of the lack of fitness costs is apparently not as critical as the non-recessive resistance. Information generated from this review suggests that planting of ‘high dose’ traits is an effective method for Bt crop IRM and more comprehensive management strategies that are also effective for functionally non-recessive resistance should be deployed.

## Supplementary Material

Supplemental Material

## Appendix

**Table A1.** Data sources and/or calculations of the dominance levels (D_FL_) of 17 cases of major resistance to Bt plants*

Case	Field resistance	No. populations investigated	Data sources and/or calculations of D’_ML_ or D’_WT_	D_FL_ measured as D’_ML_ or D’_WT_
**Resistance to single-gene Bt plants**				
*B. fusca* to Cry1Ab maize in S. Africa	Yes	1	Based on neonate-to-pupa survivorships on whole maize plants, D’_ML_was estimated to be 1.56.^33^	D’_ML_ = 1.56
*S. frugiperda* to Cry1F maize in Brazil	Yes	5	Five Brazilian populations were evaluated in three studies. Based on neonate-to-adult survivorships of a population (BR25R) on maize leaf tissue, Farias et al.^59^ reported a D’_ML_ of 0.15 for BR25R. Leite *et al*.^35^ assessed D’_ML_ of two populations (IrmaF and IrmaD) on maize leaf tissue. Based on a ‘fitness index’, which was calculated using a formula: fitness index = (neonate-to-pupal survivorship x pupal weight)/neonate-to-pupal development time), D’_WT_s of the two populations were estimated to be 0.36. In addition, Santos-Amaya *et al*.^36^ examined D’_WT_s of two other populations (MTH and MRH) using the ‘fitness index’ as described in Leite *et al*.^35^ The estimated D’_WT_ was 0.12 for MTH and 0.17 for MRH. Thus, the average D’_FL_ of the five populations was 0.23.	Mixed D_ML_ and D’_WT_ = 0.23
*S. frugiperda* to Cry1F maize in the U.S.	Yes	2	D’_ML_s of two populations, one from Puerto Rico (PR) and another from Florida (FL) were evaluated based on 7-d larval survivorship on maize leaf tissue in two studies^33,37.^ D’_ML_ of PR was 0.23 and 0.12 in two Cry1F maize hybrids, respectively with an average of 0.18.^37^ D’_ML_s estimated in the study^33^ was 0.07 for both PR and FL. D_ML_ for PR was calculated based on the average D’_ML_s of the two studies, which was 0.13. Thus, D’_ML_ for this case was calculated as the average D’_ML_ of the two populations, which was 0.10.	D’_ML_ = 0.10
*D. virgifera virgifera* to Cry3Bb1 maize in U.S.	Yes	5	For this case, five populations were evaluated in four studies. Based on neonate-to-3^rd^ instar survivorship of a population on maize seedling mat, Petzold-Maxwell et al.^39^ reported a D’_ML_ of 0.51. Ingber and Gassmann^40^ evaluated the survival-to-adult of two populations (Hopkinton and Cresco) on seedling-mat and reported a D’_ML_ of 0.37 for Hopkinton and 0.27 for Cresco. Paolino and Gassmann^41^ tested the survival-to-adult of another two populations (Elma and Monona) on seedling mat and 14-d survivorship of Monona with single- plant assays. The results showed a D’_ML_ of 0.29 for Elma and 0.45 for Monona on seedling mats, and 0.73 for Monona in single-plant assays. The average D’_ML_ of Monona in the two assay methods was 0.59. Thus, the average D’_ML_ for the five populations was estimated to be 0.41.	D’_ML_ = 0.41
*D. virgifera virgifera* to eCry3.1Ab maize in U.S.	Yes	1	Geisert *et al*.^42^ evaluated the survival of an eCry3.1Ab-resistant population using 10-d seedling bioassays and reported a D’_ML_ of 0.94 and 1.38 for the two reciprocal crosses and thus the average D’_ML_ of this case was 1.16.	D’_ML_ = 1.16
*S. frugiperda* to Cry1A.105 maize in the U.S.	Yes	2	Niu *et al*.^43^ estimated D’_ML_ based on a 7-d survivorship of two populations (RR32 and RR67) on maize leaf tissue and reported a D’_ML_ of 0.58 for RR32 and 0.10 for RR67. Thus, the D’_ML_ for this case was calculated as the average D’_ML_ of the two populations, which was 0.34.	D’_ML_ = 0.34
*O. nubilalis* to Cry1F maize in U.S.	No	1	Based on the combined data of survivorship and weight gain after 15-d release of neonates on maize plants, D’_WT_ was estimated to be 0.07 in vegetative plant stages and 0.00 in reproductive stages.^44^ Thus, D’_WT_ for this case was calculated as the average D’_WT_ (0.04) of the two test methods.	D’_WT_ = 0.04
*S. frugiperda* to Cry2Ab2 maize in U.S.	No	1	Acharya *et al*.^45^ estimated D’_ML_ based on a 7-d survivorship of a population on maize leaf tissue and reported a D’_ML_ of −0.02.	D’_ML_ = −0.02
*S. frugiperda* to Vip3A maize in Brazil	No	2	Bernatdi *et al*.^46^ evaluated D’_WT_ of a population on both whole maize plants and maize leaf tissue, and reported D’_WT_ = 0. Miraldo *et al*.^47^ evaluated neonate-to-4^th^ instar survivorship of another population on maize plants and reported D’_ML_ = 0. Thus, D’_WT_ of the case was zero.	D’_WT_ = 0.00
*S. frugiperda* to Vip3A maize in U.S	No	1	Yang *et al*.^48^ estimated D’_ML_ based on 7-d survivorship of a population on maize leaf tissue and reported a D’_ML_ of 0.00.	D’_ML_ = 0.00
*H. armigera* to Cry1Ac cotton in Australia	No	2	Bird and Akhurst^49^ examined D’_WT_ of a population based on intrinsic rate of population increase, r_m,_ on 4-week old cotton and reported a D’_WT_ of zero. In addition, based on the r_m_ values presented in Table 3 in the reference,^50^ D’_WT_ was recalculated by the author of this review and resulted in a D’_WT_ of 0.68 in Exp 1 and 0.63 in Exp 2, and thus the average D’_WT_ on 14-week cotton was 0.65. Finally, the D’_WT_ for this case was calculated as the average D’_WT_ of the two studies, which was 0.33.	D’_WT_ = 0.33
*P. gossypiella* to Cry1Ac cotton in U.S.	No	1	Liu *et al*.^51^ examined D’_ML_ based on 54-d survivorship of a population on cotton and reported a D’_ML_ of zero.	D’_ML_ = 0.00
*D. saccharalis* to Cry1Ab maize in U.S.	No	1	Wu et al.^52^ examined the 21-d larval survival of a population on seven Cry1Ab hybrids at vegetative and reproductive plant stages n the greenhouse in 2005 and 2006, respectively. D’_ML_ calculated based on the published data ranged from 0.04 to 0.28 with an average of 0.17. Ghimire *et al*.^32^ conducted two greenhouse trials and evaluated larval survivorship of a population on six Cry1Ab maize hybrids/lines and reported D’_ML_s ranged from 0.25 to 0.69 with an average of 0.42. Wangila *et al*.^53^ conducted two trials in 2010 and 2011 and evaluated larval survivorship of the same population on Cry1Ab maize plants and reported a D’_ML_ from 0.50 to 0.78 with an average of 0.65. Thus, D’_ML_ for this case was calculated as the average D’_ML_ of the three studies, which was 0.41.	D’_ML_ = 0.40
**Resistance to dual/multiple-gene Bt plants**				
*S. frugiperda* to Cry1A.105/Cry2Ab maize in Brazil	No	2	Santos-Amaya *et al*.^55^ evaluated neonate-to-adult survivorship of a population on dual-gene Cry1A.105/Cry2Ab2 maize plants and leaf tissue, and reported a D_ML_ of zero for the resistance. In addition, Horikoshi *et al*.^54^ evaluated 7-d larval survivorship of another population on maize leaf tissue and also reported a D’_ML_ of zero.	D’_ML_ = 0.00
*S. frugiperda* to Cry1A.105/Cry2Ab maize in U.S	No	1	Niu *et al*.^56^ conducted two trials and evaluated net reproductive rate (R_0_) on whole maize plants and leaf tissue, and reported a pooled D_WT_ of 0.12. Zhu *et al*.^57^ evaluated 14-d survival of the same population on whole plants and reported a D’_ML_ of 0.27. Thus, D’_FL_ for this case was calculated as the average D’_WT_ and D’_ML_ of the two studies, which was 0.20.	Mixed D’_WT_ and D’_ML_ = 0.20
*S. frugiperda* to Cry1Ab/Vip3A maize in Brazil	No	1	Horikoshi *et al*.^54^ evaluated 7-d larval survival of a population on maize leaf tissue and reported a D’_ML_ of zero.	D’_ML_ = 0.00
*S. frugiperda* to Cry1A.105/Cry2Ab2/Cry1F in Brazil	No	2	Horikoshi *et al*.^54^ and Bernardi *et al*.^58^ evaluated 7-d larval survival on maize leaf tissue and both studies reported a D’_ML_ of zero.	D’_ML_ = 0.00

* All references cited in Table A1 are listed in the main article. In addition, dominance levels of a Cry2Ab2-resistant population of *D. saccharalis* were also evaluated based on 7-d survivorship rates on leaf tissue of a Cry2Ab2 maize experimental line.^88^,The Cry2Ab2-resostant strain was documented to process a major resistance allele to the experimental Cry2Ab2 maize line. However, the experimental line used in the study expressed a relatively low level of Cry2Ab2 protein (FH personal communication), and thus, the case of the Cry2Ab2 resistance in *D. saccharalis* was excluded in this review. Larval development and survivorship of a field-collected (GA) and a Cry1Ac-selected (GA-R) populations, and their F1 progeny of *Helicoverpa zea* have been evaluated on plant tissues of non-Bt, Cry1Ac, and pyramided Cry1Ac/Cry2Ab cotton.^89,90^ Because GR was collected from Cry1Ab maize plants and it had shown significant resistance ratios to both Cry1Ac and Cry2Ab (e.g. 55-fold to Cry1Ac and 15-fold to Cry2Ab2, relative to a laboratory strain), this case was also excluded in this review.

**Table A2.** Index of fitness costs (IFC) of 20 cases of major resistance to Bt plants in eight target species.*

Case	Field resistance	No. population investigated	Sources and calculation of fitness parameters	F_S’S’_	F_R’R’_	F_R’S’_	IFC_R’R’_	IFC_R’S’_
**Resistance to single-gene Bt plants**								
*B. fusca* to Cry1Ab maize in South Africa	Yes	1	Progeny production of field-collected populations (F0) = number of eggs x egg hatching rate in Table 1 in reference^68^	151.1	213.8	n/a	1.41	n/a
		1	Fitness index = 31-d survival rate x larval mass of F1 populations in Table 2 in reference^68^	5.83	11.55	n/a	1.98	n/a
			61-d survival rate of F1 populations in Table 2 in reference^68^	0.12	0.34	n/a	2.83	n/a
			Average IFC of the two measurements for F1				2.41	n/a
			Average IFC of F0 and F1 for the case	– -	– -	– -	1.91	n/a
*S. frugiperda* to Cry1F maize in Brazil	Yes	1 (MTH)	Neonate-to-adult survivorship in Fig 4 in reference^36^	0.40	0.37	0.35	0.93	0.88
		1 (MRH)	Neonate-to-adult survivorship in Fig 4 in reference^36^	0.42	0.37	0.38	0.88	0.90
		1(IrmaF)	14-d larval survivorship in Fig 3 in reference^35^	0.71	0.72	0.73	1.01	1.03
		1	7-d larval survivorship on maize in Fig 1 in reference^54^	0.83	0.92	0.98	1.11	1.18
			7-d larval survivorship on cotton in Fig 2 in reference^54^	0.86	0.64	0.86	0.74	1.00
			Average IFC of the two test methods in the study^54^				0.93	1.09
			Average IFC of the four populations for the case				0.94	0.98
*S. frugiperda* to Cry1F maize in U.S.	Yes	1 (PR)	Fitness index = (larval survivorship x number of egg masses x egg hatching rate)/(neonate-to-adult developmental time) on maize leaf tissue in Figs 1 and 4, and Table 1 in reference^69^	0.098	0.080	0.087	0.82	0.89
			Fitness index = (larval survivorship x number of egg masses x egg hatching rate)/(neonate-to-adult developmental time) on cotton leaf tissue in Figs 1, 4, 5 and Table 1 in reference^69^	0.055	0.048	0.054	0.87	0.98
			Average of the PR population based on the study^69^				0.85	0.94
		1 (PR)	Fitness index = (neonate-to-adult survivorship x pupal mass)/neonate-to-adult developmental time in Tables 1 and Tables 2 in the reference^70^	8.35	5.35	7.62	0.64	0.91
		1(US)	Fitness index = (neonate-to-adult survivorship x pupal mass)/neonate-to-adult developmental time in Tables 1 and Tables 2 in reference^70^	8.35	4.51	8.0	0.54	0.96
			Average of the three populations for the case				0.68	0.94
*D. virgifera virgifera* to Cry3Bb1 maize in U.S.	Yes	1	Population growth rate per generation in Table 1 in reference^73^	84.00	55.80	n/a	0.66	n/a
		1	Fitness index = number of egg x hatching rate x neonate-to-adult survivorship in Table 1 (moderate selected) in reference^72^	n/a	n/a	n/a	1.28	n/a
		1	Fitness index = number of egg x hatching rate x neonate-to-adult survivorship in Table 1 (intense selected) in reference^72^	n/a	n/a	n/a	1.22	n/a
		1	Fitness index = (relative fecundity x relative egg viability x relative survivorship to adult) in Table 4 in reference^71^	1.00	1.22	n/a	1.22	n/a
		1(Hopkinton)	Fitness index = (egg production x egg viability x rate of larval survival to adult)/days of development to adults in Fig. 3 in reference^40^	51.35	55.18	n/a	1.07	n/a
		1(Cresco)	Fitness index = (egg production x egg viability x rate of survival to adult)/days of development to adults in Fig. 4 in inference^40^	11.46	4.32	n/a	0.38	n/a
		1 (Monona)	Fitness index = (egg production x egg viability x rate of survival to adult)/days of development to adults in Fig 3 in reference^41^	29.15(1210)	42.77 (1779)	n/a	1.47	n/a
		1 (Elma in the test with high food availability)	Fitness index = (egg production x egg viability x rate of survival to adult)/days of development to adults in Fig 4 in reference^41^	24.64	33.85	n/a	1.37	n/a
			Average of the 8 populations for the case			n/a	1.08	n/a
*D. virgifera virgifera* to eCry3.1Ab maize in U.S.	Yes	1	Fitness index = (larval recovery rate in 20 d x number of eggs produced per female x egg viability) in Figs 3 and 4 in reference^74^	7180	11753	n/a	1.64	n/a
*S. frugiperda* to Cry1A.105 maize in U.S.	Yes	1(RR32)	Fitness index = (neonate-to-adult survivorship x number of eggs per female)/neonate-to-adult developmental time in Table 5 in reference^43^	15.64	21.31	15.59	1.36	1.00
		1(RR67)	Fitness index = (neonate-to-adult survivorship x number of eggs per female)/neonate-to-adult developmental time in Table 5 in reference^43^	15.64	30.22	46.65	1.93	2.98
			Average of the two populations for the case				1.65	1.99
*O. nubilalis* to Cry1F maize in U.S.	No	1	Fitness index = neonate-to-adult survivorship x fertile eggs produced per female x adult mating success rate on three maize lines in Fig 3 and Table 3 in reference^75^	57.6	44.4	55.5	0.77	0.96
*S. frugiperda* to Cry2Ab2 maize in U.S.	No	1	Fitness index = (egg production x neonate-to-pupal survivorship)/neonate-to-pupal developmental time in Table 3 in reference^45^	1.34	2.51	3.20	1.87	2.39
*S. frugiperda* to Vip3A maize in Brazil	No	1	Intrinsic rate of increase (r_m_) in Table 5 in reference^46^	0.20	0.16	0.19	0.80	0.95
*S. frugiperda* to Vip3A maize in U.S.	No	1	Intrinsic rate of increase (r_m_) on maize in Fig 8 in reference^76^	0.19	0.16	0.20	0.84	1.05
			Intrinsic rate of increase (r_m_) on cotton in Fig 8 in reference^76^	0.14	0.17	0.15	1.21	1.07
			Average of the two measurements for the case				1.03	1.06
*H. armigera* to Cry1Ac cotton in Australia	No	1	Pupation rate on cotton plants reported in reference^77^	0.83	0.60	n/a	0.72	n/a
		1	Average intrinsic rate of increase (r_m_) of experiments 1 and 2 in Table 6 in reference^49^	0.20	0.14	0.19	0.70	0.95
			Average of the two studies for the case				0.71	0.95
*H. armigera* to Cry2Ab cotton in China	No	1	Neonate-to-pupal survivorship in Table 3 in reference^78^	0.30	0.23	n/a	0.77	n/a
*P. gossypiella* to Cry1Ac cotton in U.S.	No	1	Fitness index = larval survivorship/development period (degree day) in Tables 2 and Tables 3 in reference^51^	0.00071	0.00034	0.00037	0.48	0.52
*D. saccharalis to Cry1Ab maize in U.S.*	No	1	Average larval survivorship after 21 d on five non-Bt maize hybrids in two trials at two plant stages in Figs 1 and 3 in reference^52^	0.37	0.40	0.40	1.08	1.08
		1	Average larval survivorship after 21 d on two non-Bt maize hybrids in two trials in Figs 2 and 3 in reference^32^	0.44	0.57	0.57	1.30	1.30
		1	Average larval survivorship after 21 d on two non-Bt maize hybrids in two trials in Figs 2 and 4 in reference^53^	0.46	0.58	0.53	1.26	1.15
			Average of the three studies for the case				1.21	1.18
*T. ni* to Cry1Ac cotton in U.S.	No	1	Intrinsic rate of increase (r_m_) on cotton leaves in Fig 1 in reference^79^	0.16	0.15	n/a	0.94	n/a
**Resistance to dual-/multiple-gene Bt plants**								
*S. frugiperda* to Cry1A.105/Cry2Ab maize in Brazil	No	1	Neonate-to-adult survivorship in Fig 3 in reference^55^	0.26	0.24	0.27	0.92	1.04
		1	7-d larval survivorship on non-Bt maize in Fig 1 in reference^54^	0.83	0.98	0.94	1.18	1.13
			7-d larval survivorship on non-Bt cotton in Fig. 2 in reference^54^	0.86	0.83	0.84	0.97	0.98
			Average in the two crops based on the study^54^				1.08	1.06
			Average of the two populations for the case				1.00	1.05
*S. frugiperda* to Cry1A.105/Cry2Ab maize in U.S	No	1	Fitness index = net reproductive rate/neonate-to-pupa developmental time in Table 2 in reference	29.5	13.9	30.6	0.47	1.04
		1	14-d larval survivorship in Fig 2 in reference	50.5	50.0	63.5	0.99	1.26
			Average of the two studies for the case				0.73	1.15
*T. ni* to Cry1Ac/Cry2A cotton in U.S.	No	1	Intrinsic rate of increase (r_m_) on cotton leaves in Fig 1 in the reference^79^	0.16	0.13	n/a	0.81	n/a
*S. frugiperda* to Cry1Ab/Vip3A maize in Brazil	No	1	7-d larval survivorship on maize in Fig 1 in reference^80^	0.83	0.81	0.88	0.98	1.06
			7-d larval survivorship on cotton in Fig. 2 in reference^54^	0.86	0.63	0.95	0.73	1.10
			Average on the two crops for the case				0.86	1.08
*S. frugiperda* to Cry1A.105/Cry2Ab2/Cry1F in Brazil	No	1	7-d larval survivorship on maize in Fig 1 in reference^54^	0.83	0.96	0.88	1.16	1.06
			7-d larval survivorship on cotton in Fig. 2 in reference^54^	0.86	0.89	0.74	1.03	0.86
			Average on the two crops based on the study^54^				1.10	0.96
		1	r_m_ in Table 3 in reference^58^	0.18	0.16	0.18	0.89	1.00
			Average of the two populations for the case				1.00	0.98

*Index of fitness cost (IFC) of a single- or dual/multiple-gene resistance was calculated using the formula: IFC_R’R’_ = F_R’R’_/F_S’S’_ and IFC_R’S’_ = F_R’S’_/F_S’S’_ Here IFC_R’R’_ and IFC_R’S’_ mean the index of fitness costs for homozygous- (R’R’) and heterozygous (R’S’) resistant genotypes, respectively. F_R’R’_ is the fitness of homozygous-resistant genotype (R’R’) on non-Bt plants; F_R’S’_ is the fitness of heterozygous genotype (R’S’) on non-Bt plants; and F_S’S’_ is the fitness of homozygous-susceptible genotype (S’S’) on non-Bt plants. If A, B, and C represent three different resistant alleles and a, b, and c refers to the three corresponding susceptible alleles of the three genes, R’R’, R’S’, and S’S’ mean AA, Aa, and aa for a single-gene resistance; AABB, AaBb, and aabb for a dual-gene resistance; or AABBCC, AaBbCc, and aabbcc for a triple-gene resistance. IFC < 1 means that there is fitness cost; IFC = 1 suggests that there is no fitness cost; IFC > 1 indicates that there is fitness advantage associated with the resistance. All references cited in Table A2 are listed in the main publication.
