# Supplementary material for: Dominance and fitness costs of insect resistance to genetically modified Bacillus thuringiensis crops
Source: GM Crops Food. 2020 Dec 31;12(1):192–211. doi: 10.1080/21645698.2020.1852065 (PMC7781549; doi:10.1080/21645698.2020.1852065)
Supplement: Supplemental Material [file KGMC_A_1852065_SM5184.docx]

**Supporting Information**

**Appendix Table S1.** Data sources and/or calculations of the dominance levels (D_FL_) of 17 cases of major resistance to Bt plants*

| Case | Field resistance | No. populations investigated | Data sources and/or calculations of D'_ML_ or D'_WT_ | D_FL_ measured as D'_ML_ or D'_WT_ |
| --- | --- | --- | --- | --- |
| **Resistance to single-gene Bt plants** | | | | |
| *B. fusca* to Cry1Ab maize in S. Africa | Yes | 1 | Based on neonate-to-pupa survivorships on whole maize plants, D'_ML_was estimated to be 1.56.^66^ | D'_ML_=1.56 |
| *S. frugiperda* to Cry1F maize in Brazil | Yes | 5 | Five Brazilian populations were evaluated in three studies. Based on neonate-to-adult survivorships of a population (BR25R) on maize leaf tissue, Farias et al.^35^ reported a D'_ML_ of 0.15 for BR25R. Leite *et al*.^46^ assessed D'_ML_ of two populations (IrmaF and IrmaD) on maize leaf tissue. Based on a ‘fitness index’, which was calculated using a formula: fitness index = (neonate-to-pupal survivorship x pupal weight)/neonate-to-pupal development time), D'_WT_s of the two populations were estimated to be 0.36. In addition, Santos-Amaya *et al*.^47^ examined D'_WT_s of two other populations (MTH and MRH) using the ‘fitness index’ as described in Leite *et al*.^46^. The estimated D'_WT_ was 0.12 for MTH and 0.17 for MRH. Thus, the average D'_FL_ of the five populations was 0.23. | Mixed D_ML_ and D'_WT_ = 0.23 |
| *S. frugiperda* to Cry1F maize in the U.S. | Yes | 2 | D'_ML_s of two populations, one from Puerto Rico (PR) and another from Florida (FL) were evaluated based on 7-d larval survivorship on maize leaf tissue in two studies^51,66^ . D'_ML_ of PR was 0.23 and 0.12 in two Cry1F maize hybrids, respectively with an average of 0.18.^51^ D'_ML_s estimated in the study ^66^ was 0.07 for both PR and FL. D_ML_ for PR was calculated based on the average D'_ML_s of the two studies, which was 0.13. Thus, D'_ML_ for this case was calculated as the average D'_ML_ of the two populations, which was 0.10. | D'_ML_ = 0.10 |
| *D. virgifera virgifera* to Cry3Bb1 maize in U.S. | Yes | 5 | For this case, five populations were evaluated in four studies. Based on neonate-to-3^rd^ instar survivorship of a population on maize seedling mat, Petzold-Maxwell et al.^67^ reported a D'_ML_ of 0.51. Ingber and Gassmann^68^ evaluated the survival-to-adult of two populations (Hopkinton and Cresco) on seedling-mat and reported a D'_ML_ of 0.37 for Hopkinton and 0.27 for Cresco. Paolino and Gassmann^69^ tested the survival-to-adult of another two populations (Elma and Monona) on seedling mat and 14-d survivorship of Monona with single- plant assays. The results showed a D'_ML_ of 0.29 for Elma and 0.45 for Monona on seedling mats, and 0.73 for Monona in single-plant assays. The average D'_ML_ of Monona in the two assay methods was 0.59. Thus, the average D'_ML_ for the five populations was estimated to be 0.41. | D'_ML_ = 0.41 |
| *D. virgifera virgifera* to eCry3.1Ab maize in U.S. | Yes | 1 | Geisert *et al*.^70^ evaluated the survival of an eCry3.1Ab-resistant population using 10-d seedling bioassays and reported a D'_ML_ of 0.94 and 1.38 for the two reciprocal crosses and thus the average D'_ML_ of this case was 1.16. | D'_ML_ = 1.16 |
| *S. frugiperda* to Cry1A.105 maize in the U.S. | Yes | 2 | Niu *et al*.^52^ estimated D'_ML_ based on a 7-d survivorship of two populations (RR32 and RR67) on maize leaf tissue and reported a D'_ML_ of 0.58 for RR32 and 0.10 for RR67. Thus, the D'_ML_ for this case was calculated as the average D'_ML_ of the two populations, which was 0.34. | D'_ML_ = 0.34 |
| *O. nubilalis* to Cry1F maize in U.S. | No | 1 | Based on the combined data of survivorship and weight gain after 15-d release of neonates on maize plants, D'_WT_ was estimated to be 0.07 in vegetative plant stages and 0.00 in reproductive stages.^71^ Thus, D'_WT_ for this case was calculated as the average D'_WT_ (0.04) of the two test methods. | D'_WT_ = 0.04 |
| *S. frugiperda* to Cry2Ab2 maize in U.S. | No | 1 | Acharya *et al*.^65^ estimated D'_ML_ based on a 7-d survivorship of a population on maize leaf tissue and reported a D'_ML_ of -0.02. | D'_ML_ = -0.02 |
| *S. frugiperda* to Vip3A maize in Brazil | No | 2 | Bernatdi *et al*.^61^ evaluated D'_WT_ of a population on both whole maize plants and maize leaf tissue, and reported D'_WT_ = 0. Miraldo *et al*.^72^ evaluated neonate-to-4^th^ instar survivorship of another population on maize plants and reported D'_ML_ = 0. Thus, D'_WT_ of the case was zero. | D'_WT_ = 0.00 |
| *S. frugiperda* to Vip3A maize in U.S | No | 1 | Yang *et al*.^74^ estimated D'_ML_ based on 7-d survivorship of a population on maize leaf tissue and reported a D'_ML_ of 0.00. | D'_ML_ = 0.00 |
| *H. armigera* to Cry1Ac cotton in Australia | No | 2 | Bird and Akhurst^49^ examined D'_WT_ of a population based on intrinsic rate of population increase, r_m,_ on 4-week old cotton and reported a D'_WT_ of zero. In addition, based on the r_m_ values presented in Table 3 in the reference^50^, D'_WT_ was recalculated by the author of this review and resulted in a D'_WT_ of 0.68 in Exp 1 and 0.63 in Exp 2, and thus the average D'_WT_ on 14-week cotton was 0.65. Finally, the D'_WT_ for this case was calculated as the average D'_WT_ of the two studies, which was 0.33. | D'_WT_ = 0.33 |
| *P. gossypiella* to Cry1Ac cotton in U.S. | No | 1 | Liu *et al*.^75^ examined D'_ML_ based on 54-d survivorship of a population on cotton and reported a D'_ML_ of zero. | D'_ML_ = 0.00 |
| *D. saccharalis* to Cry1Ab maize in U.S. | No | 1 | Wu et al.^76^ examined the 21-d larval survival of a population on seven Cry1Ab hybrids at vegetative and reproductive plant stages n the greenhouse in 2005 and 2006, respectively. D'_ML_ calculated based on the published data ranged from 0.04 to 0.28 with an average of 0.17. Ghimire *et al*.^32^ conducted two greenhouse trials and evaluated larval survivorship of a population on six Cry1Ab maize hybrids/lines and reported D'_ML_s ranged from 0.25 to 0.69 with an average of 0.42. Wangila *et al*.^77^ conducted two trials in 2010 and 2011 and evaluated larval survivorship of the same population on Cry1Ab maize plants and reported a D'_ML_ from 0.50 to 0.78 with an average of 0.65. Thus, D'_ML_ for this case was calculated as the average D'_ML_ of the three studies, which was 0.41. | D'_ML_ = 0.40 |
| **Resistance to dual/multiple-gene Bt plants** | | | | |
| *S. frugiperda* to Cry1A.105/Cry2Ab maize in Brazil | No | 2 | Santos-Amaya *et al*.^78^ evaluated neonate-to-adult survivorship of a population on dual-gene Cry1A.105/Cry2Ab2 maize plants and leaf tissue, and reported a D_ML_ of zero for the resistance. In addition, Horikoshi *et al*.^60^ evaluated 7-d larval survivorship of another population on maize leaf tissue and also reported a D'_ML_ of zero. | D'_ML_ = 0.00 |
| *S. frugiperda* to Cry1A.105/Cry2Ab maize in U.S | No | 1 | Niu *et al*.^42^ conducted two trials and evaluated net reproductive rate (R_0_) on whole maize plants and leaf tissue, and reported a pooled D_WT_ of 0.12. Zhu *et al*.^79^ evaluated 14-d survival of the same population on whole plants and reported a D'_ML_ of 0.27. Thus, D'_FL_ for this case was calculated as the average D'_WT_ and D'_ML_ of the two studies, which was 0.20. | Mixed D'_WT_ and D'_ML_ = 0.20 |
| *S. frugiperda* to Cry1Ab/Vip3A maize in Brazil | No | 1 | Horikoshi *et al*.^60^ evaluated 7-d larval survival of a population on maize leaf tissue and reported a D'_ML_ of zero. | D'_ML_ = 0.00 |
| *S. frugiperda* to Cry1A.105/Cry2Ab2/Cry1F in Brazil | No | 2 | Horikoshi *et al*.^60^ and Bernardi *et al*.^73^ evaluated 7-d larval survival on maize leaf tissue and both studies reported a D'_ML_ of zero. | D'_ML_ = 0.00 |

* All references cited in Table S1 are listed in the main article. In addition, dominance levels of a Cry2Ab2-resistant population of *D. saccharalis* were also evaluated based on 7-d survivorship rates on leaf tissue of a Cry2Ab2 maize experimental line.^88^ The Cry2Ab2-resostant strain was documented to process a major resistance allele to the experimental Cry2Ab2 maize line. However, the experimental line used in the study expressed a relatively low level of Cry2Ab2 protein (FH personal communication), and thus, the case of the Cry2Ab2 resistance in *D. saccharalis* was excluded in this review. Larval development and survivorship of a field-collected (GA) and a Cry1Ac-selected (GA-R) populations, and their F1 progeny of *Helicoverpa zea* have been evaluated on plant tissues of non-Bt, Cry1Ac, and pyramided Cry1Ac/Cry2Ab cotton.^89,90^ Because GR was collected from Cry1Ab maize plants and it had shown significant resistance ratios to both Cry1Ac and Cry2Ab (e.g. 55-fold to Cry1Ac and 15-fold to Cry2Ab2, relative to a laboratory strain), this case was also excluded in this review.

**References**

88. Huang F, Chen M, Gowda A, Clark TL, McNulty BC, Yang F, Niu Y (2015) Identification, inheritance, and fitness costs of Cry2Ab2 resistance in a field-derived population of sugarcane borer, *Diatraea saccharalis* (F.) (Lepidoptera: Crambidae). J Invertebr Pathol 130:116−123.

89. Brévaulta T, Heuberger S, Zhang M, Ellers-Kirk C, Ni X, Masson L, Li X, Tabashnik BE Carrière Y (2013) Potential shortfall of pyramided transgenic cotton for insect resistance management. Proc Nat Acad Sci USA 110: 5806−5811

90. Carrière Y, DeGain B, Unnithan GC, Harpold VS, Li X, Tabashnik BE, (2019) Seasonal Declines in Cry1Ac and Cry2Ab Concentration in Maturing Cotton Favor Faster Evolution of Resistance to Pyramided Bt Cotton in Helicoverpa zea (Lepidoptera: Noctuidae). J Econ Entomol 112: 2019, 2907–2914.

**Appendix Table S2.** Index of fitness costs (IFC) of 20 cases of major resistance to Bt plants in eight target species.*

| Case | Field resistance | No. population investigated | Sources and calculation of fitness parameters | F_S'S'_ | F_R'R'_ | F_R'S'_ | IFC_R'R'_ | IFC_R'S'_ |
| --- | --- | --- | --- | --- | --- | --- | --- | --- |
| **Resistance to single-gene Bt plants** | | | | | | | | |
| *B. fusca* to Cry1Ab maize in South Africa | Yes | 1 | Progeny production of field-collected populations (F0) = number of eggs x egg hatching rate in Table 1 in reference^44^ | 151.1 | 213.8 | n/a | 1.41 | n/a |
|  |  | 1 | Fitness index = 31-d survival rate x larval mass of F1 populations in Table 2 in reference^44^ | 5.83 | 11.55 | n/a | 1.98 | n/a |
|  |  |  | 61-d survival rate of F1 populations in Table 2 in reference^44^ | 0.12 | 0.34 | n/a | 2.83 | n/a |
|  |  |  | Average IFC of the two measurements for F1 |  |  |  | 2.41 | n/a |
|  |  |  | Average IFC of F0 and F1 for the case | --- | --- | --- | 1.91 | n/a |
| *S. frugiperda* to Cry1F maize in Brazil | Yes | 1 (MTH) | Neonate-to-adult survivorship in Fig 4 in reference^47^ | 0.40 | 0.37 | 0.35 | 0.93 | 0.88 |
|  |  | 1 (MRH) | Neonate-to-adult survivorship in Fig 4 in reference^47^ | 0.42 | 0.37 | 0.38 | 0.88 | 0.90 |
|  |  | 1(IrmaF) | 14-d larval survivorship in Fig 3 in reference^46^ | 0.71 | 0.72 | 0.73 | 1.01 | 1.03 |
|  |  | 1 | 7-d larval survivorship on maize in Fig 1 in reference^60^ | 0.83 | 0.92 | 0.98 | 1.11 | 1.18 |
|  |  |  | 7-d larval survivorship on cotton in Fig 2 in reference^60^ | 0.86 | 0.64 | 0.86 | 0.74 | 1.00 |
|  |  |  | Average IFC of the two test methods in the study^60^ |  |  |  | 0.93 | 1.09 |
|  |  |  | Average IFC of the four populations for the case |  |  |  | 0.94 | 0.98 |
| *S. frugiperda* to Cry1F maize in U.S. | Yes | 1 (PR) | Fitness index = (larval survivorship x number of egg masses x egg hatching rate)/(neonate-to-adult developmental time) on maize leaf tissue in Figs 1 and 4, and Table 1 in reference^80^ | 0.098 | 0.080 | 0.087 | 0.82 | 0.89 |
|  |  |  | Fitness index = (larval survivorship x number of egg masses x egg hatching rate)/(neonate-to-adult developmental time) on cotton leaf tissue in Figs 1, 4, 5 and Table 1 in reference^80^ | 0.055 | 0.048 | 0.054 | 0.87 | 0.98 |
|  |  |  | Average of the PR population based on the study^80^ |  |  |  | 0.85 | 0.94 |
|  |  | 1 (PR) | Fitness index = (neonate-to-adult survivorship x pupal mass)/neonate-to-adult developmental time in Tables 1 and 2 in the reference^81^ | 8.35 | 5.35 | 7.62 | 0.64 | 0.91 |
|  |  | 1(US) | Fitness index = (neonate-to-adult survivorship x pupal mass)/neonate-to-adult developmental time in Tables 1 and 2 in reference^81^ | 8.35 | 4.51 | 8.0 | 0.54 | 0.96 |
|  |  |  | Average of the three populations for the case |  |  |  | 0.68 | 0.94 |
| *D. virgifera virgifera* to Cry3Bb1 maize in U.S. | Yes | 1 | Population growth rate per generation in Table 1 in reference^82^ | 84.00 | 55.80 | n/a | 0.66 | n/a |
|  |  | 1 | Fitness index = number of egg x hatching rate x neonate-to-adult survivorship in Table 1 (moderate selected) in reference^64^ | n/a | n/a | n/a | 1.28 | n/a |
|  |  | 1 | Fitness index = number of egg x hatching rate x neonate-to-adult survivorship in Table 1 (intense selected) in reference^64^ | n/a | n/a | n/a | 1.22 | n/a |
|  |  | 1 | Fitness index = (relative fecundity x relative egg viability x relative survivorship to adult) in Table 4 in reference^43^ | 1.00 | 1.22 | n/a | 1.22 | n/a |
|  |  | 1(Hopkinton) | Fitness index = (egg production x egg viability x rate of larval survival to adult)/days of development to adults in Fig. 3 in reference^68^ | 51.35 | 55.18 | n/a | 1.07 | n/a |
|  |  | 1(Cresco) | Fitness index = (egg production x egg viability x rate of survival to adult)/days of development to adults in Fig. 4 in inference^68^ | 11.46 | 4.32 | n/a | 0.38 | n/a |
|  |  | 1 (Monona) | Fitness index = (egg production x egg viability x rate of survival to adult)/days of development to adults in Fig 3 in reference^69^ | 29.15(1210) | 42.77 (1779) | n/a | 1.47 | n/a |
|  |  | 1 (Elma in the test with high food availability) | Fitness index = (egg production x egg viability x rate of survival to adult)/days of development to adults in Fig 4 in reference^69^ | 24.64 | 33.85 | n/a | 1.37 | n/a |
|  |  |  | Average of the 8 populations for the case |  |  | n/a | 1..08 | n/a |
| *D. virgifera virgifera* to eCry3.1Ab maize in U.S. | Yes | 1 | Fitness index = (larval recovery rate in 20 d x number of eggs produced per female x egg viability) in Figs 3 and 4 in reference^83^ | 7180 | 11753 | n/a | 1.64 | n/a |
| *S. frugiperda* to Cry1A.105 maize in U.S. | Yes | 1(RR32) | Fitness index = (neonate-to-adult survivorship x number of eggs per female)/neonate-to-adult developmental time in Table 5 in reference^52^ | 15.64 | 21.31 | 15.59 | 1.36 | 1.00 |
|  |  | 1(RR67) | Fitness index = (neonate-to-adult survivorship x number of eggs per female)/neonate-to-adult developmental time in Table 5 in reference^52^ | 15.64 | 30.22 | 46.65 | 1.93 | 2.98 |
|  |  |  | Average of the two populations for the case |  |  |  | 1.65 | 1.99 |
| *O. nubilalis* to Cry1F maize in U.S. | No | 1 | Fitness index = neonate-to-adult survivorship x fertile eggs produced per female x adult mating success rate on three maize lines in Fig 3 and Table 3 in reference^84^ | 57.6 | 44.4 | 55.5 | 0.77 | 0.96 |
| *S. frugiperda* to Cry2Ab2 maize in U.S. | No | 1 | Fitness index = (egg production x neonate-to-pupal survivorship)/neonate-to-pupal developmental time in Table 3 in reference^65^ | 1.34 | 2.51 | 3.20 | 1.87 | 2.39 |
| *S. frugiperda* to Vip3A maize in Brazil | No | 1 | Intrinsic rate of increase (r_m_) in Table 5 in reference^61^ | 0.20 | 0.16 | 0.19 | 0.80 | 0.95 |
| *S. frugiperda* to Vip3A maize in U.S. | No | 1 | Intrinsic rate of increase (r_m_) on maize in Fig 8 in reference^62^ | 0.19 | 0.16 | 0.20 | 0.84 | 1.05 |
|  |  |  | Intrinsic rate of increase (r_m_) on cotton in Fig 8 in reference^62^ | 0.14 | 0.17 | 0.15 | 1.21 | 1.07 |
|  |  |  | Average of the two measurements for the case |  |  |  | 1.03 | 1.06 |
| *H. armigera* to Cry1Ac cotton in Australia | No | 1 | Pupation rate on cotton plants reported in reference^85^ | 0.83 | 0.60 | n/a | 0.72 | n/a |
|  |  | 1 | Average intrinsic rate of increase (r_m_) of experiments 1 and 2 in Table 6 in reference^49^ | 0.20 | 0.14 | 0.19 | 0.70 | 0.95 |
|  |  |  | Average of the two studies for the case |  |  |  | 0.71 | 0.95 |
| *H. armigera* to Cry2Ab cotton in China | No | 1 | Neonate-to-pupal survivorship in Table 3 in reference^86^ | 0.30 | 0.23 | n/a | 0.77 | n/a |
| *P. gossypiella* to Cry1Ac cotton in U.S. | No | 1 | Fitness index = larval survivorship/development period (degree day) in Tables 2 and 3 in reference^75^ | 0.00071 | 0.00034 | 0.00037 | 0.48 | 0.52 |
| *D. saccharalis to Cry1Ab maize in U.S.* | No | 1 | Average larval survivorship after 21 d on five non-Bt maize hybrids in two trials at two plant stages in Figs 1 and 3 in reference^76^ | 0.37 | 0.40 | 0.40 | 1.08 | 1.08 |
|  |  | 1 | Average larval survivorship after 21 d on two non-Bt maize hybrids in two trials in Figs 2 and 3 in reference^32^ | 0.44 | 0.57 | 0.57 | 1.30 | 1.30 |
|  |  | 1 | Average larval survivorship after 21 d on two non-Bt maize hybrids in two trials in Figs 2 and 4 in reference^77^ | 0.46 | 0.58 | 0.53 | 1.26 | 1.15 |
|  |  |  | Average of the three studies for the case |  |  |  | 1.21 | 1.18 |
| *T. ni* to Cry1Ac cotton in U.S. | No | 1 | Intrinsic rate of increase (r_m_) on cotton leaves in Fig.1 in reference^87^ | 0.16 | 0.15 | n/a | 0.94 | n/a |
| **Resistance to dual-/multiple-gene Bt plants** | | | | | | | | |
| *S. frugiperda* to Cry1A.105/Cry2Ab maize in Brazil | No | 1 | Neonate-to-adult survivorship in Fig 3 in reference^78^ | 0.26 | 0.24 | 0.27 | 0.92 | 1.04 |
|  |  | 1 | 7-d larval survivorship on non-Bt maize in Fig. 1 in reference^60^ | 0.83 | 0.98 | 0.94 | 1.18 | 1.13 |
|  |  |  | 7-d larval survivorship on non-Bt cotton in Fig. 2 in reference^60^ | 0.86 | 0.83 | 0.84 | 0.97 | 0.98 |
|  |  |  | Average in the two crops based on the study^60^ |  |  |  | 1.08 | 1.06 |
|  |  |  | Average of the two populations for the case |  |  |  | 1.00 | 1.05 |
| *S. frugiperda* to Cry1A.105/Cry2Ab maize in U.S | No | 1 | Fitness index = net reproductive rate/neonate-to-pupa developmental time in Table 2 in reference | 29.5 | 13.9 | 30.6 | 0.47 | 1.04 |
|  |  | 1 | 14-d larval survivorship in Fig 2 in reference | 50.5 | 50.0 | 63.5 | 0.99 | 1.26 |
|  |  |  | Average of the two studies for the case |  |  |  | 0.73 | 1.15 |
| *T. ni* to Cry1Ac/Cry2A cotton in U.S. | No | 1 | Intrinsic rate of increase (r_m_) on cotton leaves in Fig.1 in the reference^87^ | 0.16 | 0.13 | n/a | 0.81 | n/a |
| *S. frugiperda* to Cry1Ab/Vip3A maize in Brazil | No | 1 | 7-d larval survivorship on maize in Fig.1 in reference^59^ | 0.83 | 0.81 | 0.88 | 0.98 | 1.06 |
|  |  |  | 7-d larval survivorship on cotton in Fig. 2 in reference^60^ | 0.86 | 0.63 | 0.95 | 0.73 | 1.10 |
|  |  |  | Average on the two crops for the case |  |  |  | 0.86 | 1.08 |
| *S. frugiperda* to Cry1A.105/Cry2Ab2/Cry1F in Brazil | No | 1 | 7-d larval survivorship on maize in Fig. 1 in reference^60^ | 0.83 | 0.96 | 0.88 | 1.16 | 1.06 |
|  |  |  | 7-d larval survivorship on cotton in Fig. 2 in reference^60^ | 0.86 | 0.89 | 0.74 | 1.03 | 0.86 |
|  |  |  | Average on the two crops based on the study^60^ |  |  |  | 1.10 | 0.96 |
|  |  | 1 | r_m_ in Table 3 in reference^73^ | 0.18 | 0.16 | 0.18 | 0.89 | 1.00 |
|  |  |  | Average of the two populations for the case |  |  |  | 1.00 | 0.98 |

*Index of fitness cost (IFC) of a single- or dual/multiple-gene resistance was calculated using the formula:

IFC_R'R'_ = F_R'R'_/F_S'S'_ and IFC_R'S'_ = F_R'S'_/F_S'S'_

Here IFC_R'R'_ and IFC_R'S'_ mean the index of fitness costs for homozygous- (R'R') and heterozygous (R'S') resistant genotypes, respectively. F_R'R'_ is the fitness of homozygous-resistant genotype (R'R') on non-Bt plants; F_R'S'_ is the fitness of heterozygous genotype (R'S') on non-Bt plants; and F_S'S'_ is the fitness of homozygous-susceptible genotype (S'S') on non-Bt plants. If A, B, and C represent three different resistant alleles and a, b, and c refers to the three corresponding susceptible alleles of the three genes, R'R', R'S', and S'S' mean AA, Aa, and aa for a single-gene resistance; AABB, AaBb, and aabb for a dual-gene resistance; or AABBCC, AaBbCc, and aabbcc for a triple-gene resistance. IFC < 1 means that there is fitness cost; IFC = 1 suggests that there is no fitness cost; IFC > 1 indicates that there is fitness advantage associated with the resistance.

All references cited in Table S2 are listed in the main publication.
